# Angiogenesis in Differentiated Placental Multipotent Mesenchymal Stromal Cells Is Dependent on Integrin α_5_β_1_

**DOI:** 10.1371/journal.pone.0006913

**Published:** 2009-10-22

**Authors:** Ming-Yi Lee, Jian-Pei Huang, Yi-Yung Chen, John D. Aplin, Yi-Hsin Wu, Chia-Yu Chen, Pei-Chun Chen, Chie-Pein Chen

**Affiliations:** 1 Department of Medical Research, Mackay Memorial Hospital, Taipei, Taiwan; 2 Division of High Risk Pregnancy, Mackay Memorial Hospital, Taipei, Taiwan; 3 Maternal and Fetal Health Research Group, University of Manchester, St Mary's Hospital, Manchester, United Kingdom; Institut Pasteur, France

## Abstract

Human placental multipotent mesenchymal stromal cells (hPMSCs) can be isolated from term placenta, but their angiogenic ability and the regulatory pathways involved are not known. hPMSCs were shown to express integrins α_v_, α_4_, α_5_, β_1_, β_3_, and β_5_ and could be induced to differentiate into cells expressing endothelial markers. Increases in cell surface integrins α_5_ and β_1_, but not α_4_, α_v_β_3_, or α_v_β_5_, accompanied endothelial differentiation. Vascular endothelial growth factor-A augmented the effect of fibronectin in enhancing adhesion and migration of differentiated hPMSC through integrin α_5_β_1_, but not α_v_β_3_ or α_v_β_5_. Formation of capillary-like structures in vitro from differentiated cells was inhibited by pre-treatment with function-blocking antibodies to integrins α_5_ and β_1_. When hPMSCs were seeded onto chick chorioallantoic membranes (CAM), human von Willebrand factor-positive cells were observed to engraft in the chick endothelium. CAMs transplanted with differentiated hPMSCs had a greater number of vessels containing human cells and more incorporated cells per vessel compared to CAMs transplanted with undifferentiated hPMSCs, and overall angiogenesis was enhanced more by the differentiated cells. Function-blocking antibodies to integrins α_5_ and β_1_ inhibited angiogenesis in the CAM assay. These results suggest that differentiated hPMSCs may contribute to blood vessel formation, and this activity depends on integrin α_5_β_1_.

## Introduction

Multipotent mesenchymal stromal cells from different adult and fetal tissues have been shown to have the potential to differentiate into endothelial cells [1]–[4]. Human placental multipotent mesenchymal stromal cells (hPMSCs) express genes associated with ectoderm, endoderm and mesoderm, including hematopoietic/endothelial cell-related transcripts [5]. They can differentiate into osteogenic, adipogenic, chondrogenic, and neurogenic cell lineages [6]–[8], and, under the combined influence of growth factors and mechanical shear stress, have been reported to acquire aspects of the endothelial phenotype [9]. However, the angiogenic ability of these cells is not well characterized.

Angiogenesis is a complex process that involves extracellular matrix (ECM) remodeling, endothelial cell differentiation, migration and proliferation, and the functional maturation of new endothelial cell colonies into mature blood vessels [10]. There is evidence that fibronectin is a key ECM component at several stages, initially providing attachment sites for precursor cells [11], [12], then promoting vascular endothelial growth factor (VEGF)-induced differentiation to endothelial cells [13]. Furthermore, fibronectin associated with VEGF–A enhances endothelial cell migration [14]. Targeted gene deletion studies have revealed that fibronectin functions in vascular stabilization and branching morphogenesis in the murine embryo [15]–[18], while a more restricted gene targeting approach that deletes alternatively spliced variants of fibronectin leads to defective placental angiogenesis [19]. Fibronectin is abundant in the mesenchymal compartment of human placenta where vasculogenesis and angiogenesis occur [20].

Integrin α_5_β_1_ is a selective high affinity receptor for fibronectin, and a regulator of VEGF-A signaling [21]. Integrin α_5_β_1_ is observed to have an essential role in pathological neovascularization in cornea [22] and is up-regulated in newly growing vessels in embryos and tumors [21], [23], [24]. Vascularization in the placenta is critical for normal delivery of nutrients to the fetus. Placental growth is most rapid in the first half of pregnancy, but development of the vascular tree continues to term [25], [26]. Placental vascular defects, including reduced vessel density, are associated with fetal growth restriction [27], [28]. VEGF-A is thought to play an important role in human placental vascularization, especially in the early stages [29].

Improved understanding of the cellular and molecular mechanisms of placental vasculogenesis and angiogenesis could potentially lead to treatments to achieve improved pregnancy outcome as well as the possibility of using placental progenitor cells in therapeutic applications. Thus, the aims of this study were to investigate if hPMSCs are capable of functional differentiation into endothelial cells, and to investigate the role in this process of integrin α_5_β_1_ and its interaction with fibronectin in the presence of VEGF-A.

## Materials and Methods

### Isolation and culture, of placenta-derived cells

Clinically normal human placentas (37 to 40 weeks of gestation, n = 30) were obtained after cesarean section. Tissue was collected after written informed consent was obtained, and this study was approved by the Institutional Review Board of Mackay Memorial Hospital, Taipei. The animal studies were specifically approved by the ethics committee of Mackay Memorial Hospital for animal experimentation and were conducted following the institution's guidelines for animal husbandry.

Isolation of hPMSCs was performed as we described previously [30]. Briefly, about 100 g of tissue from central placental cotyledons was minced, trypsinized (0.05% trypsin-EDTA solution; Invitrogen), and treated with 10 U/ml DNase I (Sigma-Aldrich) in Dulbecco's modified Eagle's medium (DMEM; Gibco) at 37°C for 5 min several times, and finally filtered through a cell strainer (BD Biosciences). The supernatants were pooled and centrifuged, and the mononuclear cells in the supernatants were recovered by Percoll density gradient fractionation (1.073 g/ml, Sigma-Aldrich) [31]. The cells were resuspended and seeded in a flask, and were maintained in DMEM with 10% fetal bovine serum (FBS; Hyclone) at 37°C in a humidified atmosphere with 5% CO_2_. Approximately 2 to 3 weeks later, some colonies containing fibroblast-like cells were observed. These cells were trypsinized and replated for expansion after 70% confluence.

### Flow cytometry

The cell phenotype of hPMSCs was characterized by a panel of PE- or FITC-conjugated antibodies purchased from Serotec, Calbiochem, Chemicon, R&D systems, GeneTex, or BD Biosciences using standard fluorescence-activated cell sorting analysis and CellQuest software. The cells were permeabilized using BD Cytofix/Cytoperm^TM^ Fixation/Permeabilization Kit (BD) according to the manufacturer′s recommendation before flow cytometry assay for Oct-4 (Chemicon).

Alterations of integrin abundance on the cell surface of hPMSCs were evaluated by fluorescence intensity, an index of the surface concentration of integrin per cell. Mean fluorescence is the estimated x axis value at maximum peak height. This parameter is measured independently from the proportion of positive cells, estimated as the difference between the mean fluorescence (the surface concentration of integrin molecules) of positive cells and that of the control samples, regardless of the frequency of positive cells [32].

### Reverse-transcription PCR (RT-PCR)

The following primers were used for PCR reactions: human Oct-4 sense, 5′-CGTGAAGCTGGAGAAGGAGAAGCTG-3′; antisense, 5′-CAAGGGCCGCAGCTTACACATGTTC-3′; homeobox transcription factor nanog (Nanog) sense, 5′-AATACCTCAGCCTCCAGCAGATG-3′; anti-sense, 5′-CTGCGTCACACCATTGCTATTCT-3′; Sry-Box 2 (Sox-2) sense 5′-GGCAGCTACAGCATGATGCAGGAGC-3′; antisense, 5′-CTGGTCATGGAGTTGTACGCAGG-3′; CD31 sense, 5′-GACGTGCTGTTTTACAACATCTC-3′; antisense, 5′-CCTCACGATCCCACCTTGG-3′; CD34 sense, 5′-GCGCTTTGCTTGCTGAGTTT-3′; antisense, 5′-GCCATGTTGAGACACAGGGT-3′; VE-cadherin sense, 5′-GGGAGACCACGCCTCTGTC-3′; antisense, 5′-GGAGGCCCTGGGCATCTC-3′; VEGFR (vascular endothelial growth factor receptor)-1 sense, 5′-GAAGGCATGAGGATGAGAGC-3′; antisense, 5′-CAGGCTCATGAACTTGAAAGC-3′; VEGFR-2 sense, 5′-GGCCAAGTGATTGAAGCAGATG-3′; antisense, 5′-TTCAGATCCACAGGGATTGCTC-3′; human von Willebrand factor (vWF) sense, 5′-ACGTGATCCTTCTCCTGGATG-3′; antisense, 5′-TTCACCACGTTGGAGTCGCCT-3′; human CD105 sense, 5′-CAACATGCAGATCTGGACCAC-3′; antisense, 5′-CTTTAGTACCAGGGTCATGGC-3′; 18S sense, 5′-TAGAGCTAATACATGCCGACGG-3′; antisense, 5′-GGGCCTCGAAAGAGTCCTGTATT-3′. The RT-PCR reactions were performed as previously described [4], [33]–[37].

### Differentiation induction

#### Induction of osteogenic or adipogenic differentiation

hPMSC cells in passages 6 through 10 (n = 5) were cultured in either osteogenic medium consisting of DMEM containing 10% FBS (Hyclone), 0.1 µM dexamethasone, 10 mM β-glycerol phosphate, 500 µM ascorbic acid (Sigma-Aldrich) or adipogenic medium consisting of DMEM containing 10% FBS (Hyclone), 1 µM dexamethasone, 500 µM isobutylmethylxanthine, 200 µM indomethacin, and 10 µg/ml insulin (Sigma-Aldrich) [4], [7]. The medium was changed every 3 days. After 2 weeks of culture, the cells were fixed with methanol, stained either with 1% Alizarin Red S (Sigma-Aldrich) to assess calcium phosphate deposition indicating osteogenic differentiation or with Oil Red O (Sigma-Aldrich) to look for lipid droplets indicating adipogenic differentiation [38].

#### Induction of endothelial cell differentiation

hPMSCs were seeded at a density of 1×10^3^ cells/cm^2^ in petri dishes and cultured in endothelial cell growth medium 2 (EGM2; Promocell) supplemented with Supplement Mix (Promocell) which contains 1 µg/ml ascorbic acid, 10 ng/ml human recombinant basic fibroblast growth factor, 5 ng/ml human recombinant epidermal growth factor, 22.5 µg/ml heparin, 0.2 µg/ml hydrocortisone, 20 ng/ml long R3 insulin like growth factor-1, 0.62 ng/ml phenol red, 0.5 ng/ml human recombinant VEGF-A, and 2% FBS (Hyclone). Additionally, 50 ng/ml VEGF-A (Chemicon) was added to medium for induction of differentiation. The cultures were maintained for 14 to 21 days and the culture medium was replaced every three days [1], [4].

### Immunofluorescence staining

Immunofluorescence staining was performed as previously described [20]. Primary antibody against specific proteins included CD31 (JC70A; 1∶50; Dako), CD34 (QBEND/10; 1∶100; Serotec), VE-cadherin (BV6; 1∶100; Chemicon), vWF (1∶200; Sigma-Aldrich), CD105 (SN6h; 1∶50; Dako), VEGFR-1 (Flt-1/EWC; 1∶1000; abcam), VEGFR-2 (89106; 1∶50; R&D Systems), integrin β_1_ (HUTS-4; 1∶1000; Chemicon), α_4_ (P4G9; 1∶1000; Chemicon), α_5_ (SAM-1; 1∶100; Chemicon), α_ν_β_3_ (LM609; 1∶100; Chemicon), and α_ν_β_5_ (P1F6; 1∶1000; Chemicon). Antibodies were diluted as appropriate in 1% BSA in PBS. 4′, 6-diamidino-2-phenylindole (DAPI; Sigma-Aldrich) was used to identify nuclei.

### Adhesion assay

The 24-well plates were coated with 10 µg/ml human fibronectin (Upstate), vitronectin (Upstate), or 2.5% BSA (as a control) at 37°C for one hour. After washing with PBS, the wells were blocked with 2.5% (wt/vol) BSA in PBS at room temperature for one hour. VEGF-A induced differentiated hPMSCs were preincubated (30 min at 37°C) in HBSS containing either 3 µg/ml non-specific mouse immunoglobulin (IgG; Dako) or a mouse blocking monoclonal antibody specific to integrin β_1_ (HUTS-4; 1∶1000; Chemicon), α_4_ (P4G9; 1∶1000; Chemicon), α_5_ (SAM-1; 1∶100; Chemicon), α_ν_β_3_ (LM609; 1∶100; Chemicon), or α_ν_β_5_ (P1F6; 1∶1000; Chemicon). A total of 6×10^4^ cells in 500 µl EGM2 supplemented with 0.2% BSA were seeded to the precoated wells and allowed to adhere for 30 min at 37°C. Adherent cells were quantified by counting in six randomly selected fields per well (magnification 200×; Axiovert 200; Carl Zeiss MicroImaging).

### Transwell migration assay

Cell migration assay were performed by 8-µm-pore transwells (Costar). The VEGF-A induced differentiated hPMSCs were preincubated (30 min at 37°C) in HBSS containing either 3 µg/ml non-specific mouse IgG (Dako) or function-blocking mouse monoclonal antibody specific to integrin β_1_ (HUTS-4; 1∶1000; Chemicon), α_4_ (P4G9; 1∶1000; Chemicon), α_5_ (SAM-1; 1∶100; Chemicon), α_ν_β_3_ (LM609; 1∶100; Chemicon), or α_ν_β_5_ (P1F6; 1∶1000; Chemicon). A total of 5 to 8×10^5^ hPMSCs were added to the upper chamber of the transwell and allowed to migrate for 20 hours at 37°C with EGM2 (600 µl; Promocell) in the presence or absence of VEGF-A (50 ng/ml) together with either 0.5 to 50 µg/ml of fibronectin (Upstate) or vitronectin (Upstate) in the lower chamber. The number of cells transmigrated to the lower compartment was quantified by counting migrated cells in six randomly selected fields per well (magnification 200×; Axiovert 200; Carl Zeiss MicroImaging).

### In vitro angiogenesis

Induction of capillary tube formation was performed using an In Vitro Angiogenesis kit (Chemicon) as recommended by the manufacturer. VEGF-A-induced differentiated hPMSCs cells were preincubated (30 min at 37°C) in HBSS containing either 3 µg/ml non-specific mouse IgG (Dako) or function-blocking monoclonal antibody specific to integrin β_1_ (HUTS-4; 1∶1000; Chemicon), α_4_ (P4G9; 1∶1000; Chemicon), α_5_ (SAM-1; 1∶100; Chemicon), α_ν_β_3_ (LM609; 1∶100; Chemicon), or α_ν_β_5_ (P1F6; 1∶1000; Chemicon). After blocking with these antibodies, cells were resuspended to 1×10^5^ cells/ml in EGM2, after which 100 µl/well of cell suspension was added onto a gel of polymerized basement membrane-like material (ECMatrix^TM^). The cells were incubated for 6 hours at 37°C for full development of capillary-like network structures. Tube formation was quantified by counting the number of polygonal tubes as well as cumulative tube length (long axis). In instances where several tube-like structures merged together or branched, the total length of the tubes was calculated as the sum of the length of the individual branches [39]. Results are represented as total tube length (µm) or number for six random photographic fields per experimental condition (magnification 50×; Axiovert 200; Carl Zeiss MicroImaging). The experiment was independently repeated three times.

### In vivo angiogenesis by chick chorioallantoic membrane assay

The chick chorioallantoic membrane (CAM) assay was modified from a prior report [40]. CAM was exposed by cutting a window (2 cm^2^) on one side of 10-day-old specific pathogen free chicken eggs. A 3-mm-thick sterile straw disk, 8 mm in diameter, was placed on the CAM for 3-dimensional culture on an area with a minimum of small blood vessels. A total of 1×10^5^ of hPMSCs pretreated either with anti-integrin β_1_ (HUTS-4; 1∶1000; Chemicon), α_4_ (P4G9; 1∶1000; Chemicon), α_5_ (SAM-1; 1∶100; Chemicon) or non-specific mouse IgG (1∶50; Dako) and resuspensed in 100 µl EGM2 were placed into the straw. The window in the shell was sealed with adhesive tape and the egg was incubated for 48 hours. Representative CAMs from each treatment group were photographed under a dissecting microscope (10×) and counted. The number of fine blood vessel branch points in the region of the sample was counted. As angiogenesis is characterized by the sprouting of new vessels in response to hPMSCs, counting blood vessel branching points is a useful quantitative means of obtaining an angiogenic index. At least 6 embryos were used per treatment group. Data were evaluated in terms of average number of blood vessel branching points per treatment group ± SD. CAMs were further excised, cryopreserved, cut into 5-µm sections, and immunostained with vWF, CD31 and CD105 as described above. For cell tracking, hPMSCs with or without induction into endothelial differentiation by VEGF-A were labeled with green fluorescence dye CellTracker^TM^ CMFDA (5 µM, Molecular Probes, Invitrogen) before implanting into CAM. The CAM was immunostained by primary antibody against human vWF (1∶800, Sigma-Aldrich) after cell transplantation. Quantification of new vessel formation that contained hPMSC incorporation or the number of hPMSCs that incorporated into the endothelia of each vessel were conducted in 10 fields (400×) for each section from 3 randomly selected sections of each CAM tissue.

### Statistical analysis

The measurements of counting were conducted blind by two independent observers. The intra-class correlation coefficient for intra- and inter-rater reliability of cell counts *>*0.75 was considered good agreement. The data are described as means ± SD. Differences were assessed using the independent-samples t test, paired-samples t test, or Mann Whitney U test when appropriate. A *P* value of less than 0.05 was considered significant. The statistical software used is SSPS version 12.0 (Chicago,IL,USA).

## Results

### Characterization of human placenta-derived multipotent mesenchymal stromal cells

**Figure 1:**
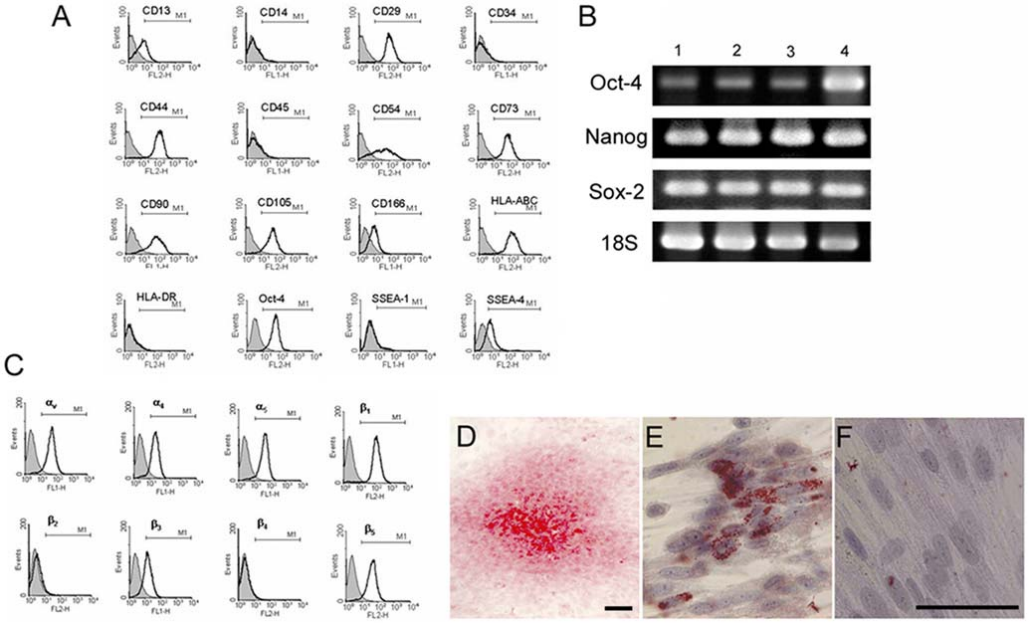
Characterization of human placental multipotent mesenchymal stromal cells (hPMSCs). (A) Flow cytometry analysis of cell-marker expression by hPMSCs. The cells express CD13, CD29, CD44, CD54, CD73, CD90, CD105, CD166, HLA-ABC, Oct-4, and SSEA-4, but are negative for HLA-DR, CD34, CD45, and SSEA-1. The shaded area shows the profile of the cells stained by a control antibody of matching isotype. Ten thousand cells were counted from each sample. (B) Analysis of stem cell markers on hPMSCs by RT-PCR analysis. Three different strains of hPMSCs are shown in lane 1 to 3. Lane 4 was RNA obtained from a human embryonic stem cell line (hES 6; ES Cell International, USA) and served as a positive control. PCR was performed for 35 cycles to demonstrate Oct-4 and Sox-2 transcripts, and 30 cycles for Nanog. (C) The expression of integrin subunits on hPMSCs determined by flow cytometry. The data shown are representative of 3 different experiments. (D) Osteogenic differentiation of hPMCs resulted in positive staining for Alizarin Red S, indicating the presence of calcium salt deposition associated with the matrix. (E) Adipocyte differentiation resulted in cytoplasmic lipid droplets that were Oil Red O–positive. (F) Undifferentiated hPMSCs used as a control for Oil Red O staining. The cells were counterstained with Mayer's hematoxylin (E, F). Scale bar: 100 µm.

Flow cytometric analysis showed that hPMSCs were positive for the multipotent mesenchymal stromal cell markers CD13, CD29, CD44, CD54, CD73, CD90, CD105, and CD166, but negative for the hematopoietic stem cell markers CD45 and CD34 and the monocytic marker CD14 (Fig. 1A). These characteristics are consistent with multipotent mesenchymal stromal cells. The cells expressed HLA-ABC (MHC I), but not HLA-DR (MHC II; Fig. 1A). hPMSCs were also positive for the embryonic stem cell-associated cell surface marker stage-specific embryonic antigen (SSEA)-4 (38.2±10.4%), and the pluripotent stem cell-specific transcription factor Oct-4 (78.4±11.5%). However, hPMSCs do not express SSEA-1 (Fig. 1A), TRA-1-60, or TRA-1-81 (not shown). The absence of CD34 in the hPMSC isolate excludes endothelial cell contamination. Flow cytometry analysis further showed hPMSCs to express integrins α_v_, α_4_, α_5_, β_1_, β_3_, and β_5_, while β_2_ and β_4_ were undetectable (Fig. 1C).

RT-PCR analysis of total RNA from cultured hPMSCs showed expression of Oct-4, Nanog and Sox-2, transcription factors associated with pluripotency [41]–[43] (Fig. 1B). hPMSC multipotency was demonstrated using standard osteocyte and adipocyte differentiation assays. In the former case hPMSCs formed nodules and stained with Alizarin Red S, indicating calcium salt crystallization and osteogenic differentiation (Fig. 1D). In the latter assay, hPMSCs developed Oil Red O–positive cytoplasmic lipid droplets indicating adipocyte differentiation (Fig. 1E). This was not seen in control cells (Fig. 1F).

### Differentiation of hPMSCs into endothelial cells

**Figure 2:**
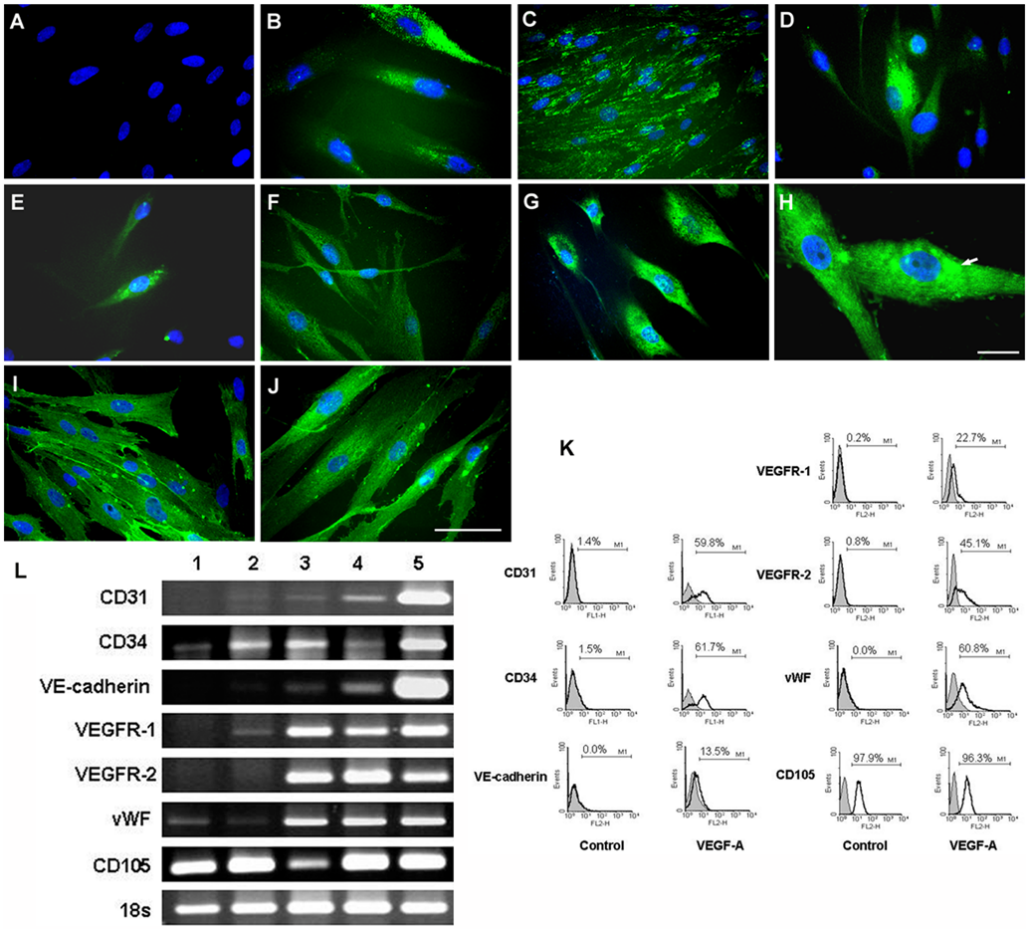
Characterization of human placental multipotent mesenchymal stromal cells (hPMSCs) after inducing differentiation into endothelial cells. Immunofluorescence staining of CD31 in undifferentiated hPMSCs is shown in (A) as a negative control. Immunofluorescence of endothelial markers in differentiated hPMSCs under induced culture conditions: (B) CD31, (C) CD34, (D) VE-cadherin, (E) VEGFR-1, (F) VEGFR-2, (G) von Willebrand factor, (H) von Willebrand factor under high magnification (Scale bar: 1 µm); Weibel-Palade bodies were visible within the differentiated hPMSC cytoplasm (arrow). CD105 was positive in (I) undifferentiated hPMSCs and (J) hPMSCs differentiated into endothelial cells. Cell nuclei were counterstained with DAPI. Scale bar: 10 µm. (K) Flow cytometry analysis of endothelial cell markers on the hPMSC surface before and after differentiation induced by VEGF-A. (L) mRNA for endothelial cell markers was amplified from 2 different strains of undifferentiated hPMSCs (lane 1 and 2) and hPMSCs differentiated into endothelial cells (lane 3 and 4). mRNA from human umbilical vein endothelial cells was used as a positive control (lane 5). The data shown are representative of 3 different experiments.

hPMSCs were cultured in the presence of EGM2 with 2% FBS and 50 ng/ml VEGF-A for 14 to 21 days, then stained with antibodies to the endothelial markers: CD31, CD34, VE-cadherin, VEGFR-1, VEGFR-2, vWF, and CD105, and assayed by flow cytometry (Fig. 2). Undifferentiated hPMSCs cultured with regular medium showed no significant immunofluorescence staining for CD31 (Fig. 2A), CD34, VE-cadherin or vWF (not shown). The overall fluorescence intensity of CD31 (Fig. 2B), CD34 (Fig. 2C), VE-cadherin (Fig. 2D), VEGFR-1, VEGFR-2 and vWF (Fig. 2E–H) in differentiated hPMSCs was markedly enhanced after 21 days of cultivation, indicating the differentiation of hPMSC to endothelial cells. Weibel-Palade bodies were clearly visible within differentiated hPMSCs under high magnification (Fig. 2H). CD105 was expressed in differentiated hPMSCs (Fig. 2J). However, the undifferentiated cells were also positive for CD105 (Fig. 2I). Flow cytometry revealed a substantial increase of expression of CD31 (3.1±1.6% vs. 56.6±11.4%; P = 0.012), CD34 (1.2±1.0% vs. 46.3±14.1%; P = 0.029), VE-cadherin (0.8±0.7% vs. 14.1±4.4%; P = 0.038), VEGFR-1 (1.5±1.1% vs. 27.0±6.2%; P = 0.015), VEGFR-2 (1.9±1.0% vs. 46.4±2.7%; P = 0.001) and vWF (0.8±0.7% vs. 63.2±7.1%; P = 0.004), but not CD105 (94.6±6.3% vs. 88.1±8.3%; P = 0.825) in differentiated hPMSCs compared to control cells (Fig. 2K).

Endothelial differentiation of hPMSCs was further revealed by mRNA analysis. Two different strains of hPMSCs were used for comparison before and after differentiation. Consistent with a previous report [5], the hPMSCs express various hematopoietic genes. Transcription from the CD34 and CD105 genes was variable between hPMSC isolates. However, after differentiation, the cells showed increased levels of mRNA encoding CD31, VE-cadherin, VEGFR-1, VEGFR-2, and vWF (Fig. 2L).

**Figure 3:**
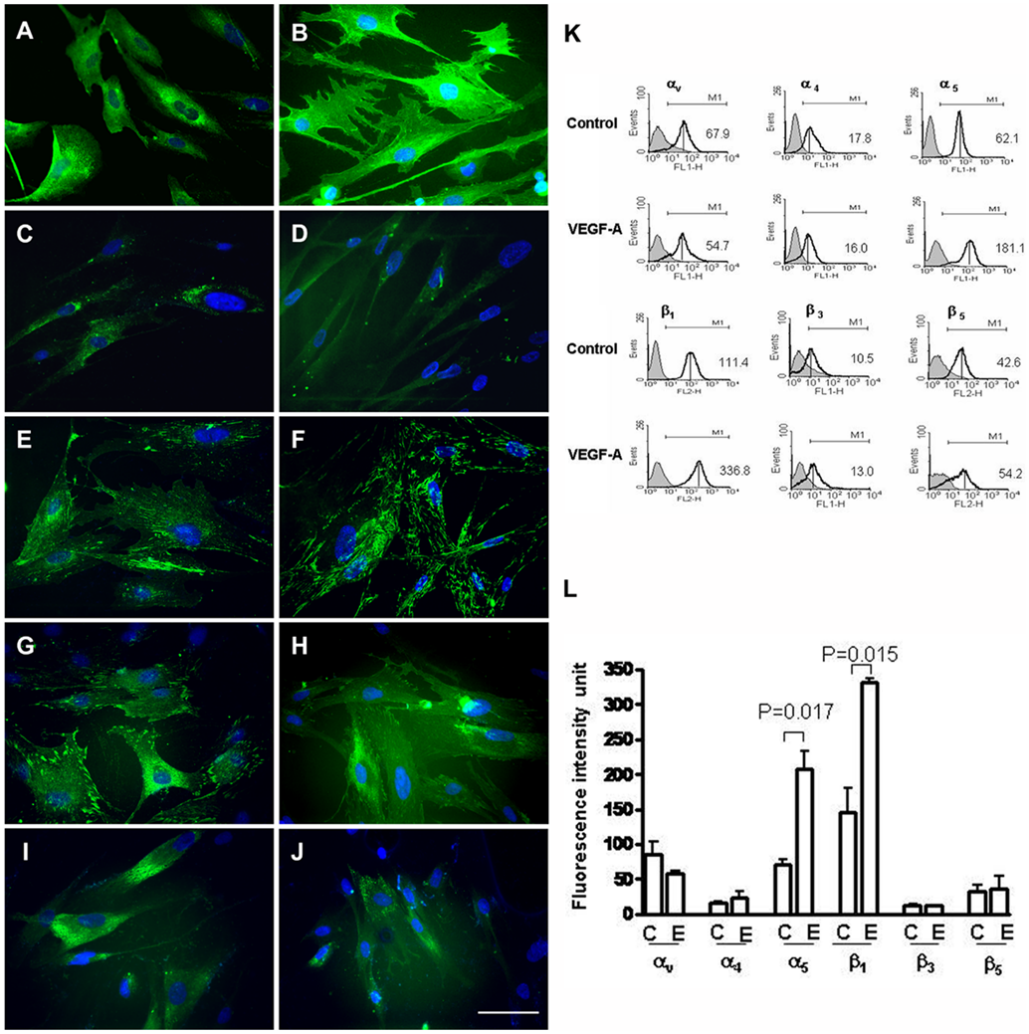
Integrin expression at the surface of human placental multipotent mesenchymal stromal cells (hPMSCs) before and after inducing differentiation into endothelial cells. Undifferentiated hPMSCs: A, C, E, G, I; differentiated hPMSCs: B, D, F, H, J. The cells were immunostained using antibody against integrin (A, B) β_1_, (C, D) α_4_, (E, F) α_5_, (G, H) α_v_β_3_, or (I, J) α_v_β_5_. Scale bar: 10 µm. Immunofluorescence staining revealed significant increases in integrin α_5_ and β_1_. (K) The change of hPMSC surface integrin expression after differentiation induced by VEGF-A was measured by flow cytometry and expressed in fluorescence intensity units. Change in fluorescence intensity is an index of integrin surface concentration per cell. (L) Quantification of specific mean fluorescence intensity (which corresponds to the increase in fluorescence intensity relative to second antibody alone) is shown (±SD, n = 3). The specific mean fluorescence intensity was higher for both integrins α_5_ and β_1_ in differentiated hPMSCs. C: controls; E: endothelial cell differentiation induced by VEGF-A.

Immunofluorescence staining demonstrated integrins α_4_, α_5_, β_1_, α_v_β_3_, and α_v_β_5_ in undifferentiated hPMSCs. Integrin α_5_ (Fig. 3E, 3F) and β_1_ (Fig. 3A, 3B) were significantly increased after endothelial differentiation, but not integrin α_4_ (Fig. 3C, 3D), α_v_β_3_ (Fig. 3G, 3H), or α_v_β_5_ (Fig. 3I, 3J). Cell surface levels of integrin α_v_, α_4_, α_5_, β_1_, β_3_, and β_5_ subunits were assayed by flow cytometry before and after endothelial differentiation. Mean specific fluorescence (which corresponds to the increase in fluorescence intensity relative to second antibody alone) was higher for both integrins α_5_ and β_1_ in differentiated hPMSCs (Fig. 3K, 3L). This suggests a significant increase in cell surface integrin α_5_β_1_ expression as the cells differentiated. In contrast, no increase was observed in expression of the αvβ3 or αvβ5 integrin, which have also been implicated in binding of fibronectin, vitronectin and other RGD–containing ligands, and in angiogenesis [21], [44], [45].

### Fibronectin and integrin α_5_β_1_ promote VEGF-A-induced differentiated hPMSC adhesion and migration

**Figure 4:**
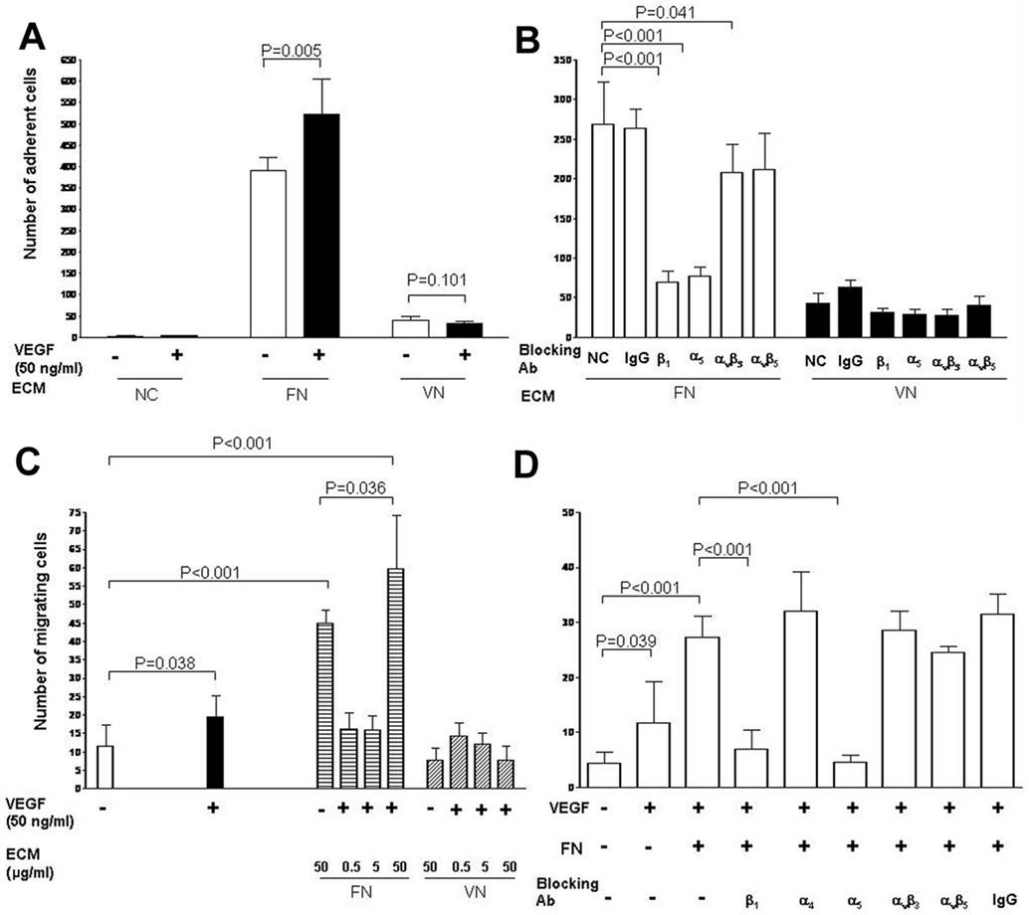
Adhesion and migration assays were performed with human placental multipotent mesenchymal stromal cells (hPMSCs) and various integrin-blocking antibodies. (A). hPMSCs were induced to differentiate into endothelial cells, then plated onto surfaces coated with BSA (NC), fibronectin, or vitronectin in the presence or absence of VEGF-A. The differentiated hPMSCs adhered to fibronectin more efficiently than to vitronectin (P<0.001). VEGF-A significantly increased the adhesion of differentiated hPMSCs to fibronectin but not to vitronectin or plates coated with 2.5% BSA solution (NC). (B) Inhibition of differentiated hPMSC adhesion to fibronectin and vitronectin in the presence of blocking antibodies to integrin subunits. A significant inhibition of differentiated hPMSC adhesion to fibronectin was observed in the presence of blocking antibodies to integrin α_5_, β_1_ or α_v_β_3_, but not integrin α_v_β_5_. The number of differentiated hPMSCs attached to vitronectin was low. (C) Transwell migration of differentiated hPMSCs induced by VEGF-A (50 ng/ml) with or without fibronectin (0.5 to 50 µg/ml) or vitronectin (0.5 to 50 µg/ml). Fibronectin in the presence of VEGF-A stimulated migration. (D) Inhibition of differentiated hPMSC transwell migration induced by VEGF-A (50 ng/ml) with or without fibronectin (50 µg/ml). Various blocking antibodies to integrin subunits and non-specific IgG were used. Antibodies to integrin subunits α_5_ and β_1_ were inhibitory. Error bar: SD; Ab: antibody; ECM: extracellular matrix.

Since integrin α_5_β_1_ is a specific receptor for fibronectin, and angiogenesis often involves endothelial cell adhesion and migration within a fibronectin-rich ECM, we investigated the ability of differentiated cells to interact with fibronectin. As the cells also express integrins α_v_β_3_ and α_v_β_5_, vitronectin was used as a control ligand. Differentiated hPMSCs adhered to fibronectin more efficiently than to vitronectin (P<0.001). VEGF-A significantly increased the adhesion of differentiated hPMSCs to fibronectin but not to vitronectin- or BSA-coated plates (Fig. 4A). Significant inhibition of differentiated hPMSC attachment to fibronectin-coated plates was observed in the presence of blocking antibodies to integrin α_5_ and β_1_, whereas very limited inhibition effect was observed in the presence of antibody to α_v_β_3_ and α_v_β_5._ Attachment to vitronectin was low, and blocking antibodies had no significant effect (Fig. 4B). These experiments indicated that integrin α_5_β_1_ on the surface of differentiated hPMSCs is important for mediating cell attachment to fibronectin, with a further contribution from α_v_β_3_.

Migration of differentiated hPMSC was enhanced by either VEGF-A (50 ng/ml) or fibronectin (50 µg/ml) alone, but the combination of VEGF-A and fibronectin produced an additive effect (Fig. 4C). In contrast, neither vitronectin alone nor the combination of VEGF-A and vitronectin promoted migration (Fig. 4C). In the presence of antibodies to integrin α_5_ or β_1_, cell migration to VEGF-A and fibronectin together was suppressed, whereas antibodies to α_4_, α_v_β_3_, or α_v_β_5_ had no effect (Fig. 4D). These results suggest strongly that enhanced adhesion and migration of differentiated hPMSC in the presence of VEGF-A and fibronectin are dependent on integrin α_5_β_1_.

### The ability of differentiated hPMSCs to form capillary-like structures is mediated by integrin α_5_β_1_

**Figure 5:**
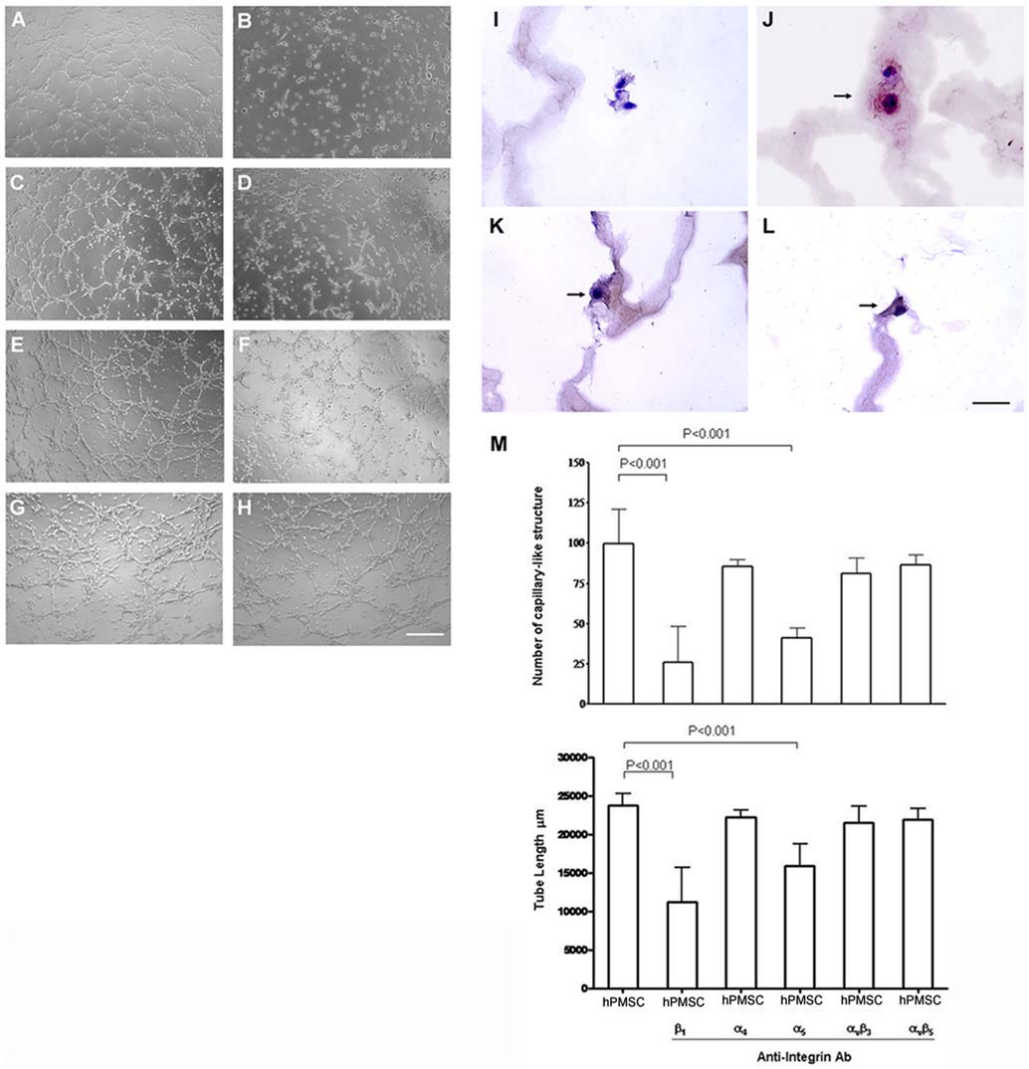
In vitro angiogenesis: formation of capillary-like structures by differentiated human placental multipotent mesenchymal stromal cells (hPMSCs). hPMSCs were trypsinized, seeded on wells coated with ECMatrix^TM^. (A) Human umbilical vein endothelial cells and (B) undifferentiated hPMSCs were used as positive and negative control (shown after 6 hours of culture). (C) hPMSCs induced to differentiate into endothelial cells form characteristic capillary-like structures. Cell elongation and cell interconnecting cell networks were observed. Differentiated hPMSCs were pretreated with antibodies against (D) integrin β_1_, (E) α_4_, (F) α_5_, (G) α_v_β_3_, or (H) α_v_β_5_. Scale bar: 200 µm. Representative photomicrographs of 3 different experiments are shown. This ability of differentiated hPMSCs to form capillary-like structures was strongly diminished when integrin β_1_ or α_5_ was inhibited. Blocking antibodies to integrins α_4_, α_v_β_3_, or α_v_β_5_ did not inhibit the formation of capillary-like structures. The capillary-like structures on ECmatrix^TM^ gel were immunostained using antibody against (I) non-specific IgG or specific endothelial markers (J) von Willebrand factor, (K) CD31, and (L) CD105. Scale bar: 50 µm. Arrows indicate von Willebrand factor, CD31 and CD105 positive cells present in ECmatrix^TM^ gel. (M) Quantification of the capillary-like structures by measuring the polygonal network (upper panel) and the cumulative tube length (lower panel) formed by differentiated hPMSCs. A significant inhibition of capillary-like structure formation was observed when integrin β_1_ or α_5_ antibody was applied to the cells. Error bar: SD.

To study the molecular mechanisms underlying capillary-like morphogenesis in differentiated hPMSCs, either differentiated hPMSCs or human umbilical vein endothelial cells (HUVECs) were seeded onto a basement membrane-like gel. HUVEC (Fig. 5A) and undifferentiated hPMSCs (Fig. 5B) were used as positive and negative controls, respectively. The undifferentiated hPMSCs showed very few capillary-like structures after 6 hours, and most cells remained rounded (Fig. 5B). The differentiated hPMSCs typically showed cytoplasmic projections, spikes and extensions, and had elongated within 6 hours, with most cells becoming integrated into capillary-like structures (Fig. 5C). Formation of these structures was strongly inhibited in the presence of antbodies to integrin β_1_ (Fig. 5D) or α_5_ (Fig. 5F). Blocking antibodies to integrin α_4_, α_v_β_3_, or α_v_β_5_ did not show inhibitory activity (Fig. 5E, 5G, 5H; quantified in Fig. 5M). Immunostaining confirmed that cells in the capillary-like structures express the specific endothelial markers including vWF (Fig. 5J), CD31 (Fig. 5K), and CD105 (Fig. 5L). Figure 5I was the cells stained by non-specific IgG as a control.

### Integrin α_5_β_1_ mediates the angiogenesis of differentiated hPMSCs in vivo

**Figure 6:**
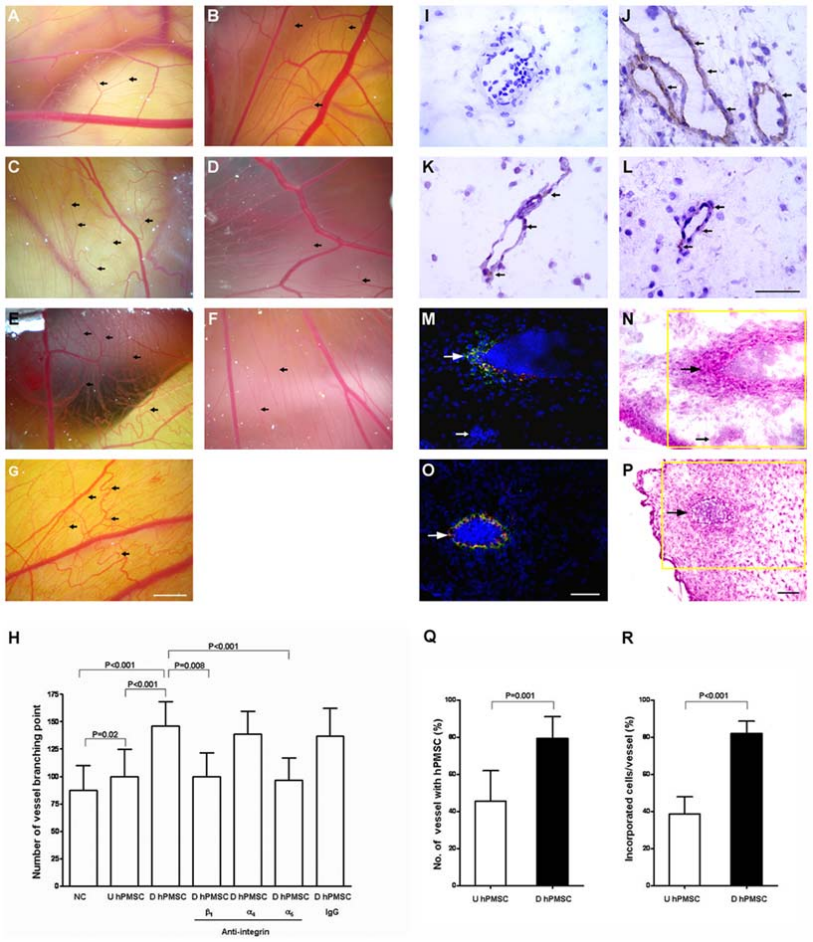
Stimulation of angiogenesis in chick chorioallantoic membrane (CAM) by undifferentiated or differentiated human placental multipotent mesenchymal stromal cells (hPMSCs). Small tortuous blood vessels, typical of neovascularization, were visible in the CAM. The development of new blood vessels was determined by counting branch points (arrows) after 48 hours of cell transplantation. Sprouting and branching vessels (arrows) are prominent in the CAM transplanted by (C) differentiated hPMSCs compared to the (A) negative control (fresh culture medium) without hPMSC transplantation and (B) undifferentiated hPMSCs transplantation. CAMs were implanted by differentiated hPMSCs pre-treated with antibodies against integrin (D) β_1_, (E) α_4_, (F) α_5_, or (G) non-specific IgG. Scale bar: 3 mm. Representative photomicrographs of 6 different chicken embryos are shown. (H) Quantification of new blood vessels formed by negative control (NC; fresh culture medium) or hPMSCs in CAM. The angiogenic effect was substantially reduced in hPMSCs pre-treated with anti-integrin α_5_ or β_1_ antibody. Cryosections of CAMs were immunostained by (I) von Willebrand factor (vWF), (J) vWF, (K) CD31, and (L) CD105 antibodies. (I) CAM without hPMSC transplantation was negative for vWF staining, indicating human vWF antibody does not cross react with CAM. (J–L) Arrows indicated vWF, CD31 and CD105-positive cells present in CAM, revealing that differentiated hPMSCs contributed to neovascularization. CAM transplanted with (M) undifferentiated hPMSCs or (O) differentiated hPMSCs were immunostained using a rhodamine (red)-conjugated human vWF antibody. The hPMCs were labeled with CellTracker^TM^ CMFDA (green). DAPI (blue) staining was used to identify nuclei. The incorporation of hPMSC into CAM endothelium that expressed vWF would reveal yellow signal. Differentiated hPMSCs were widely incorporated into endothelium of CAM vessels (O). A large arrow indicates the hPMSCs incorporated into CAM vessel endothelium. A small arrow indicates a nearby vessel lacking both engrafted hPMSCs and human vWF expression (M). Plenty of nucleated red blood cells were observed within the lumens of vessels. (N, P) Hematoxylin-eosin stained serial sections with inset areas matching images of M and O. Scale bar: 50 µm. (Q) Quantification of the number of new vessels formation that contained hPMSCs after transplantation of differentiated or undifferentiated hPMSCs. (R) Quantification of the number of hPMSCs incorporated into vessel endothelium after transplantation of differentiated or undifferentiated hPMSCs. D hPMSCs: differentiated hPMSCs; U hPMSCs: undifferentiated hPMSCs; Error bar: SD.

Differentiated or undifferentiated hPMSCs were implanted onto CAMs of ten-day-old chick eggs, and two days later the CAM was imaged (Fig. 6A–G) and vessel branching points counted (Fig. 6H). In contrast to the short-term incubation in vitro, the transplanted undifferentiated hPMSCs augmented angiogenesis (Fig. 6B, 6H). However, there was a statistically significant increase of neovascularization in the CAM transplanted by differentiated hPMSCs compared to that of undifferentiated hPMSCs or control (Fig. 6A–C and 6H). The angiogenic activity of the differentiated hPMSCs was significantly reduced by preincubation with anti-integrin α_5_ or β_1_ prior to transfer to the CAM (Fig. 6D and 6F). Addition of anti-integrin α_4_ or non-specific mouse IgG antibody had no significant effect on angiogenic activity (Fig. 6E and 6G). Immunostaining of the CAM further revealed cells within the neovessels were positive for human vWF, CD31 and CD105 (Fig. 6J–L). The CAM without hPMSC transplantation was vWF negative (Fig. 6I). These results demonstrated that the differentiated hPMSCs contributed to the neovascularization of CAM and the angiogenic activity is mediated through integrin α_5_ and β_1_.

To assess hPMSC distribution after transplantation, differentiated hPMSCs were found distributed widely in CAM vasculature (Fig. 6O, 6Q). Immunofluorescence demonstrated a part of these incorporated hPMSCs labeled with CellTracker^TM^ CMFDA expressed endothelial cell-specific protein vWF (Fig. 6M, 6O). A significantly higher number of vessels of CAM contained hPMSCs after transplantation with differentiated hPMSCs compared to undifferentiated cells (79.4±11.7% vs. 45.5±16.5%; P = 0.001; Fig. 6Q), and differentiated hPMSCs were significantly more numerous in the vessels than undifferentiated cells (81.1±8.2% vs. 38.7±9.2%; P<0.001; Fig. 6R).

## Discussion

hPMSCs have the potential to differentiate into endothelial cells under appropriate conditions both in vitro and in vivo. The differentiated cells express a panel of endothelial cell makers including vWF, CD31, CD34, VE-cadherin and the endothelial cell receptors VEGFR-1 and VEGFR-2 in vitro and in vivo. They can form capillary tube-like structures in vitro and have a greater capacity to augment angiogenesis in the CAM assay than undifferentiated cells. The ability of differentiated hPMSCs to form new blood vessels involves integrin β_1_ and α_5_, but not integrin α_v_, α_4_, β_3_, or β_5_.

In agreement with previous reports [5]–[8], the phenotype of hPMSC is similar to that of human bone marrow-derived multipotent mesenchymal stromal cells [31], [46]. Although undifferentiated hPMSCs express various hematopoietic gene transcripts [5], they do not bear the hematopoietic marker CD45. They express adhesion molecules and mesenchymal stem cell markers, including CD29, CD44, CD73, CD105, and CD166 [47], [48]. We also observed that hPMSCs express genes that are considered to be specific to pluripotent embryonic cells [41]–[43] such as Oct-4 and SSEA-4. SSEA-4 was previously thought to mark specifically human embryonic stem cells, but has also been observed in subpopulations of adult bone marrow-derived or placenta-derived multipotent mesenchymal stromal cells [6], [49], [50].

Fibronectin and VEGF-A are important regulators of blood vessel growth [51], [52]. Fibronectin is highly expressed within the hematopoietic microenvironment [53] and is involved in the adhesion of hematopoietic stem and progenitor cells [12], [54], [55]. Fibronectin acts as a ligand for integrins α_5_β_1_, α_4_β_1_ and α_v_β_3_ [21], [44], [56]–[58]. However, hPMSC adhesion to, and migration on fibronectin are specifically dependent on subunits β_1_ and α_5_, the expression of both subunits increases upon endothelial differentiation, and angiogenesis is inhibited by blocking either of these two subunits. Hence we suggest the heterodimer α_5_β_1_ interacts with fibronectin in the pericellular matrix to mediate key steps in angiogenesis. VEGF-A and fibronectin together significantly promote the adhesion and migration of hPMSCs. This augmentation effect is specific to fibronectin and the α_5_β_1_ integrin.

Alterations of integrin expression may contribute to angiogenesis. It has been found that VEGF-A increases the migration of human dermal microvascular endothelial cells through the upregulation of α_v_β_3_ integrin expression [59]. Cells undergoing a TGF-β-induced angiogenic program up-regulate integrin α_5_ [60]. A significant up-regulation of α_5_ subunit expression in vascular cells participating in choroidal neovascularization of injured eye was observed [61]. Furthermore, during the early phase of vessel sprouting, when interacting with fibronectin-rich interstitial ECM, activated endothelial cells may utilize integrin α_5_β_1_ or α_v_β_3_ to mediate the angiogenic response, later switching to other integrin subunits once basement membrane ligands such as laminin have assembled around the new vessel [62]–[65]. These reports suggest that α_5_β_1_ or α_v_β_3_ may play a role in neovascularization and provide a target for therapeutic intervention [61], [66]. We observed that VEGF-A increased the expression of integrin α_5_β_1_, but not α_v_β_3_ or α_v_β_5_.

Targeted gene ablation reveals that successful vasculogenesis depends on integrin α_5_β_1_ [18] and its ligand fibronectin [16], [67], and is not strongly dependent on integrin α_v_β_3_ [18]. Homozygotic integrin α_5_-deficient mouse embryos demonstrate vascularization disruption and die in utero with numerous morphological defects [15]. Fibronectin-deficient mice also develop defects in the yolk sac vasculature [16], [67]. In integrin α_5_-null embryonic cells, development of a complex vasculature is hindered with reduced cell proliferation and increased apoptosis [17]. Integrin β_1_ is required for the initiation of basement membrane formation. In integrin β_1_-null embryonic bodies, the complex vasculature formation is significantly delayed [68]. In contrast, vasculogenesis and angiogenesis in virtually all organs develop normally in α_v_-null embryos [69]. Mice lacking β_3_ integrins or both β_3_ and β_5_ integrins not only enhance tumor growth, but have enhanced angiogenesis. Thus, neither β_3_ nor β_5_ integrins are essential for neovascularization [70]. Furthermore, both β_3_-null and β_5_-null mice are viable, with unaffected developmental angiogenesis and adult angiogenesis such as retinal neovascularization and wound healing [71], [72]. These reports support our observations that angiogenesis by differentiated hPMSCs is independent of integrin α_v_, β_3_ and β_5_.

In contrast to the short incubation period of in vitro angiogenesis assays, both undifferentiated and differentiated hPMSCs were observed to enhance angiogenesis in the CAM assay. hPMSCs with positive vWF staining were observed to engraft in the endothelium of CAM. Direct integration of hPMSCs into CAM vasculature may augment sprouting angiogenesis. Similar to the study of ischemic brain or heart, multipotent mesenchymal stromal cells widely incorporated into vasculature and a subset of them was capable of differentiating into endothelial cells [73], [74]. However, hPMSCs after inducing endothelial cell differentiation have more cell numbers incorporated into CAM vessel and have a significantly greater angiogenic effect than that of undifferentiated hPMSC. Additionally, we cannot exclude other specific adhesion molecules or growth factors which may regulate distinct angiogenic responses. The paracrine factors expressed by hPMSCs may participate in enhancing angiogenesis other than the transdifferentiation into endothelial cells by hPMSCs [75].

Our results are the first to identify a role for integrins in the regulation of angiogenesis initiated by hPMSCs. hPMSCs are a useful model to study the role of VEGF-A in differentiation and maturation of endothelial cells, and the role of integrins during placental angiogenesis and vasculogenesis. Transplantation of hPMSCs may offer potential for treating ischemic diseases. Transplantation of hPMSCs to the ischemic limbs of SCID mice significantly improved blood vessel formation and blood flow in the affected limbs [76]. In addition these cells could be utilized in the engineering of complex tissues in whcih vascularization is an essential feature.
